# Emerging role of the RNA-editing enzyme ADAR1 in stem cell fate and function

**DOI:** 10.1186/s40364-023-00503-7

**Published:** 2023-06-06

**Authors:** Di Lu, Jianxi Lu, Qiuli Liu, Qi Zhang

**Affiliations:** The Biotherapy Center, the Third Affiliated Hospital of Sun Yat-Sen University, Guangzhou, 510630 China

**Keywords:** Adenosine deaminase acting on RNA-1 (ADAR1), Stem cells, Cell fate, RNA editing

## Abstract

Stem cells are critical for organism development and the maintenance of tissue homeostasis. Recent studies focusing on RNA editing have indicated how this mark controls stem cell fate and function in both normal and malignant states. RNA editing is mainly mediated by adenosine deaminase acting on RNA 1 (ADAR1). The RNA editing enzyme ADAR1 converts adenosine in a double-stranded RNA (dsRNA) substrate into inosine. ADAR1 is a multifunctional protein that regulate physiological processes including embryonic development, cell differentiation, and immune regulation, and even apply to the development of gene editing technologies. In this review, we summarize the structure and function of ADAR1 with a focus on how it can mediate distinct functions in stem cell self-renewal and differentiation. Targeting ADAR1 has emerged as a potential novel therapeutic strategy in both normal and dysregulated stem cell contexts.

## Introduction

Stem cells have self-renewal and multidirectional differentiation potential. According to the different developmental stages, they are divided into embryonic stem cells (ESCs) and adult stem cells (ASCs) [1, 2]. Stem cell therapy has prominent application prospects. For example, allogenic hematopoietic stem cell (HSC) transplantation can rebuild the patients’ hematopoietic and immune system, and mesenchymal stem cells (MSCs) can reduce transplantation rejection and severe COVID-19-induced lung injury [3–6]. Under the accumulation of gene mutations, cell fusion, chromosomal mutations, etc., mechanisms regulating the self-renewal and differentiation potential of the stem cells are compromised on, which then drive tumor growth from the malignant transformation of stem cells. Numerous studies have shown that the pluripotency, differentiation ability and cell reprogramming of stem cells are regulated by various transcription factors, such as Oct4, Sox2, Nanog, Klf4 and c-Myc [7, 8]. However, recent studies have found that posttranscriptional modifications are critical for regulating different cellular processes and stem cell fate [9–12]. Posttranscriptional modifications have been increasingly demonstrated to be important for both RNA biosynthesis and degradation, including N6-methyladenosine (m^6^A), *N*^*1*^-methyladenosine (m^1^A), inosine (I), pseudouridine (Ψ), 5-methylcytosine (m^5^C), 5-hydroxymethylcytosine (5-hmC), *N6,2’-O*-dimethyladenosine (m^6^A_m_), and 7-methylguanosine (m^7^G) [13–18]. The modification of mRNA widely affects key biological processes, such as its folding [19], translation [20] and transport [21, 22]. In recent years, A-to-I editing, which is the most common type of RNA editing in animals, has gradually attracted attention. It is widely involved in a variety of gene regulatory mechanisms at the transcriptional and posttranscriptional levels, including alteration of sequences coding for amino acids at the transcriptome level and mRNA splicing, mRNA stability and circular RNA formation. Its dysregulation drives aberrant transcription and translation programs that promote cancer occurrence and progression [23–25].

As an enzyme acting on double-stranded RNA (dsRNA), the ADAR protein family was discovered in 1987 [26–28]. The ADAR family has three different members: ADAR1, ADAR2 and ADAR3. ADAR1 and ADAR2 have enzymatic activities that explain the existence of RNA editing in different tissues [29–31], while ADAR3 is only present in brain tissue and has no enzymatic activity, which may compete for dsRNA substrates and thus act as an RNA editing inhibitor in the brain [32]. ADAR1, a specific adenosine deaminase, binds to dsRNA and converts adenosine (A) to inosine (I) after RNA transcription, which is known as A-to-I editing [33]. It contains three isoforms, p150, p110 and p80 [34, 35]. P150 has been reported to be involved in the regulation of type I interferon signaling [36]. The IFN-inducible p150 heterodimer of ADAR1 contains a Zα structural domain that recognizes RNA with an alternative left-handed double helix structure, termed Z-RNA. Heterozygous ADAR1 mutations in the Zα structural domain cause type I IFN-mediated disorders in humans and mice. Mutations in the ADAR1 gene are associated with Aicardi-Goutières syndrome (AGS) and Dyschromatosis symmetrica hereditaria (DSH), where AGS manifests mainly as neurological lesions associated with chronic activation of type I interferon (IFN) [37]. Analysis of ADAR1 mutations in AGS patients showed 11 mutations, with 8 amino acid substitutions located in the catalytic domain with a significant increase in the production of interferon-α [37, 38]. Like AGS, DSH is characterized by exceeding 130 ADAR1 mutations. Both DSH and AGS share a common mutation, Gly1007Arg. This is also the only missense mutation that completely eliminates the editing activity of ADAR1 [38–40]. Moreover, ADAR1 blocks endogenous Z-RNA-dependent activation of Z-DNA binding protein 1(ZBP1) in response to pathogenic type I IFN, suggesting that ZBP1 may be a key molecule in type I interferon disease caused by ADAR1 mutations [41–43].

ADAR1 has been found to affect immune cell functions, for instance, mediating early T-cell development [44] and T-cell immune tolerance and preventing colitis [45, 46]. ADAR1 is involved in regulating macrophage function and maintaining the balance of DC cell subsets [47–49].

In addition to its classical role in triggering adaptive immunity, there has been a growing report about the effects of ADAR1 on stem cells. A study found that knockout of ADAR1 leads to lethality or premature death of mouse embryos, suggesting that RNA editing regulation has vital biological significance [50]. ADAR1 promotes leukemia stem cell (LSC) self-renewal via let-7 pri-microRNA editing [51] ADAR1 knockdown also reduces the self-renewal ability of blast crisis leukemia stem cell (BC-LSC) in RAG2^+^ γc^+^ mice. These data show that ADAR1 reprogram malignant progenitor cells to drive leukemia progression [52]. By the way, ADAR1 plays an important role in the survival and maintenance of intestinal stem cells and intestinal homeostasis by inhibiting endoplasmic reticulum (ER) stress and interferon (IFN) signal transduction. ADAR1 is highly expressed in Lgr5^+^ cells, and its absence in adult mice leads to rapid apoptosis and loss of these active circulating stem cells in the small intestine and colon [53]. In addition, the Wnt/β-catenin pathway can be triggered by ADAR1, thereby affecting the regulation of the proliferation of malignant hematopoietic stem cells [51, 54, 55]. However, the specific mechanism of ADAR1 as a new target for the clinical treatment of stem cell-related diseases is not yet known.

In this context, we will discuss the protein structure and biological function of ADAR1, and its potential role in stem cell. In addition, we describe some theoretically feasible treatment strategies for stem cells related diseases based on ADAR1 function in this review.

## Structure and molecular function of ADAR1

### Structure and expression of ADAR1

**Fig. 1:**
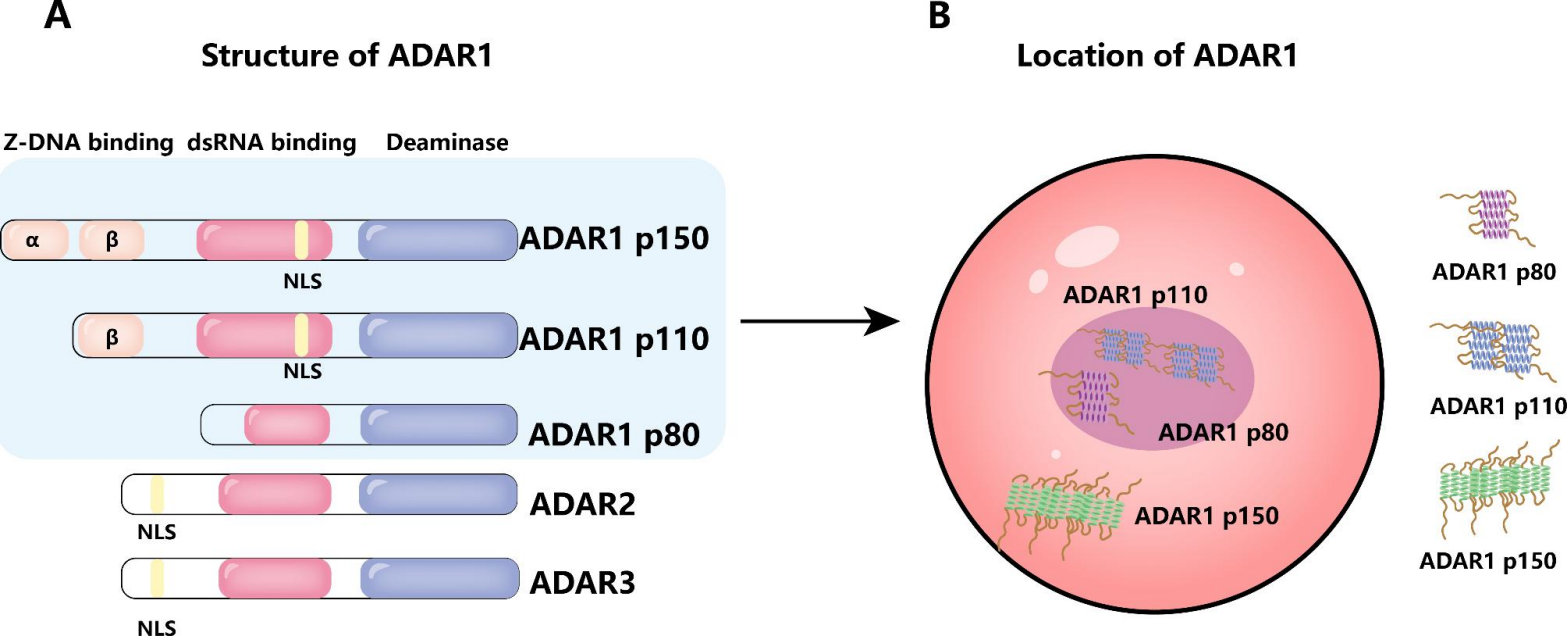
**(A)** Structure of ADAR1. **(B)** Location of ADAR1. ADAR1 p150 is located in the cytoplasm, while ADAR1 p110 is mainly located in the nucleus. Additionally, ADAR1 p80 exits the nucleus

The human ADAR1 gene is located at the long arm of chromosome 1 (1q21.3), and its three isoforms have different protein structures but are arranged by similar domains. ADAR1 p150 has the longest protein structure, starting from the N-terminus, and there are two domains, α and β, as Z-DNA binding domains, wherein the Zα domain contains the nuclear export sequence (NES) [56]. It also contains three double-stranded RNA (dsRNA) binding domains, and the last dsRNA binding domain contains a nuclear localization sequence (NLS) [57]. There is a lengthy deaminase activity domain at the C-terminal, which performs the function of enzymatic activity [58]. Compared to ADAR1 p150, ADAR1 p110 lacks the Zα domain and contains a NLS in the third dsRNA-binding domain [56]. The presence of NLS and NES can explain why ADAR1 p150 is mainly located in the cytoplasm but can shuttle between the nucleus and cytoplasm. In contrast, ADAR1 p110 is mainly localized in the nucleus [58]. The two ADAR1 isoforms, p150 and p110, are formed from different promoters and alternate splicing. Among them, ADAR1 p150 includes interferon-stimulated response element (ISRE) and it is the only one induced by interferon (IFN), which is different from the constitutive expression of ADAR1 p110[59, 60]. Another variant of ADAR1 exists, called p80. ADAR1 p80 is localized in the nucleolus and starts at methionine 519 (M519), which deletes the putative NLS, the Z-DNA binding domain and the entire RNA binding domain due to alternative splicing of exon 2. Since ADAR1 heterodimers are differentially regulated during acute inflammation, it is suggested that the localization of these variants and A-to-I RNA editing in the cytoplasm, nucleus and nucleolus are reorganized after an intracellular response to inflammatory stimuli [35]. (Fig. 1)

### Molecular functions of ADAR1

**Fig. 2:**
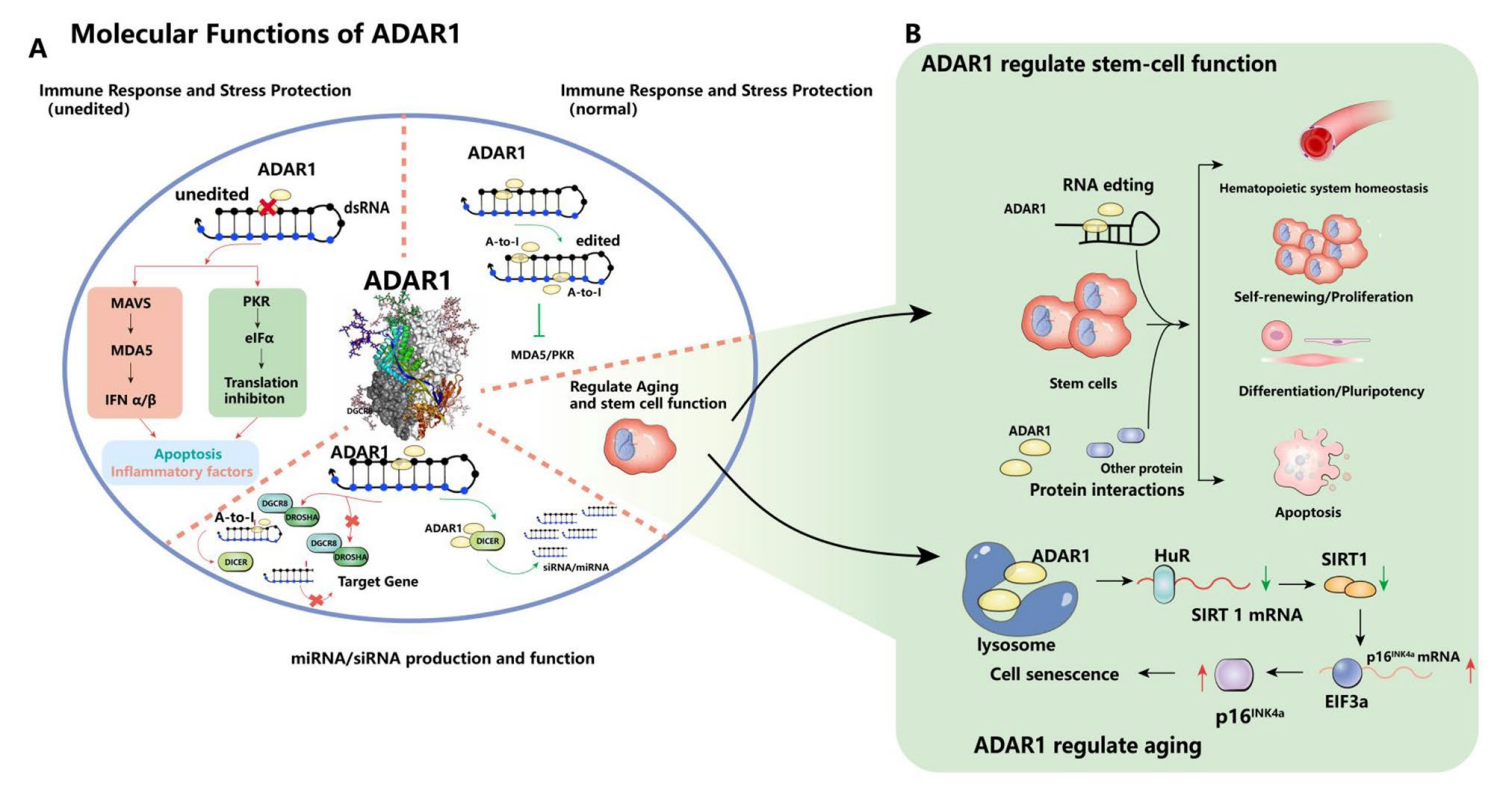
**(A)** The role of ADAR1 in miRNA production and the immune response. ADAR1 editing of mRNA does not activate downstream pathways under normal conditions; however, when it is absent, it causes activation of the MDA5 and PKR pathways, leading to apoptosis. ADAR1 can not only inhibit miRNA production but also alter its targeting to cause changes in target genes. In addition, ADAR1 can promote the production of miRNAs by forming complexes. **(B)** Molecular functions of ADAR1 in regulating stem cells and aging. ADAR1 affects stem cell function and cell fate through three pathways. The pathways include affecting senescence, immune response and miRNA production. In addition, the cells regulate ADAR1 through autophagy, which leads to the decline of *SIRT1* mRNA stability and up-regulation of downstream p16 protein expression, and finally cause cell aging

ADAR1 editing functions to convert the hydrolytic deamination of nucleotides from adenosine (A) to inosine (I). A-to-I editing is mainly found in noncoding regions and is most prevalent in *Alu* repeat elements throughout the human genome [57]. The double-stranded RNA (dsRNA) sequence is formed by the strong complementarity of the *Alu* sequence in close proximity. A high-throughput detection analysis of the A-to-I editing site revealed that the *Alu* element is the largest source of endogenous dsRNA in human cells [61]. On ADAR mediated RNA editing, translation machinery reads inosine as guanine and pairs with cytosine, altering protein function during protein coding and regulating protein diversity [23]. In addition, ADAR1 exhibits some regulatory ability under physiological conditions, such as the immune response and aging [62, 63]. (Fig. 2)

#### miRNA/siRNA production and function

ADAR1 homodimers edit dsRNA or the precursors of microRNA (miRNA), and the edited dsRNA cannot be recognized and cleaved by Drosha or Dicer enzymes due to structural changes, which reduces the number of miRNAs or siRNAs [64, 65].

Nevertheless, some studies have shown that ADAR1 can promote the production of miRNA or siRNA in addition to antagonizing the effect of RNAi. In this case, ADAR1 abandons its original sister partner and forms a heterodimer with the Dicer enzyme, which enhances targeted mRNA degradation by promoting the cleavage efficiency of the Dicer enzyme to generate more miRNA and siRNA [66] (Fig. 2A).

*Alu* is the most frequently occurring retrotransposon in the human genome in the form of a tandem repeat. Since *Alu* repeats frequently occur within the 3’-UTR of the coding region where miRNAs bind, editing of *Alu* sequences has the potential to alter or create new miRNA targets [67–69]. ADAR1 changes the miRNA binding site by editing the 3’-UTR, thereby causing abnormal expression of cancer-related genes. For example, when A-to-I editing occurs at the 3’-UTR of MDM2, miR-155, which is responsible for gene silencing, cannot bind to it, so that the mRNA of MDM2 remains stable and its downstream p53 is inactivated [70]. Interestingly, ADAR1 functions in an editing-independent manner in the 3’-UTR. It binds nascent transcripts for instance in the 3’-UTR to inverted repeat elements where it interferes with other RNA-processing factors [67]. Nishikura K. et al. demonstrated that ADAR1 significantly promote the processing of *Alu* dsRNA into *Alu* siRNA. And this *Alu* endo siRNA targets protein 1 (CDCP1) mRNA containing the CUB domain, which contains an antisense copy of *Alu*Jb in its 3 ‘UTR [67, 71]. Moreover, knocking down ADAR1 in gastric cells leads to a decrease in miR-148a-3p, which ultimately leads to a decrease in mRNA and protein levels of nuclear transcription factor Y subunit α (NFYA), proving ADAR1’ editing-independent ability [72].

Thus, ADAR1 can regulate the expression of target miRNA/siRNA through editing-dependent and editing-independent pathways (Table 1**).**

**Table 1:** The downstream targets (mRNA/miRNA) of ADAR1 and its biological functions

Target	Mechanism of ADAR1 action		Biological functions
AZIN1 [136]		mRNA editing in 3’-UTR	Influence hematopoietic stem cell self-renewal and differentiation
CCDC15 [137]		Edit a GA-rich ISS at intron 8 of CCDC15	Affect *CCDC15* splicing Promote tumorigenesis
CCN1[120]		mRNA editing	Induce immune response As a tumor-suppressor in mutiple cancers
DHFR [138]		mRNA editing in 3’-UTR	Enhance cellular proliferation and resistance to methotrexate in breast cancer resistance
FAK [139]		Editing a specific intronic region	Facilitate tumor progression and metastasis
GLI1[111, 140]		mRNA editing in exon 12	Increase proportion of drug-resistant cells in myeloma
Let-7 [51, 141]		Editing in pri-let-7d	Impair pri-let-7 biogenesis Promote leukemia stem cell (LSC) self renewal
miR-222[141, 142]		ADAR1p150 complex with Dicer	Promote the expression of miRNA-222 Induce Myocarditis
miR-378a-3p [119]		miRNA editing	Prevent melanoma progression
miR-455-5p [142]		miRNA editing	Promotes melanoma growth and metastasis
c-Myc [125]		Stabilize c-Myc mRNA	Promote resistance of pancreatic cancer cells to BET inhibitors
MDM2[70]		Editing the 3’ UTR of MDM2	Promote malignant progenitor propagation
miR-148a-3p [72]		Together with DICER enerates endogenous small RNAs	Correlate with the development of gastric cancer
p16^INK4a^ [63]		Affect SIRT1 RNA stability through HuR	Promote senescence
miR-26a [36]		Impairing miR-26a maturation,	Represses CDKN1A expression accelerates cell cycle transit
GM2A [105]		Editing 3’-UTR of GM2A	Confer GSC self-renewal and stemness
EMT miRNAs [143–145]		miRNA editing	Promote DICER-mediated miRNA maturation Promote cell invasion and migration

#### Immune response and stress protection

Seeburg PH. et al. explained that the mouse embryo of E12.5 is lethal after ADAR1 knockout attribute to the rapid disintegration of liver structure and severe defects in ultimate hematopoiesis, including developmental disorders such as erythroid and myeloid/granulocyte progenitor cells, as well as splenic colony formation and activity in the aorta gonad mesonephric region and fetal liver [50]. A study by Mannion’s group suggests that ADAR1 plays a crucial role in interferon-stimulated genes (ISGs) and interferon signaling [73]. Besides, ADAR1 inhibits the MDA5/MAVS signaling pathway and rescues apoptosis and embryonic death [62, 74, 75]. ADAR1 specifically regulates the MDA5-MAVS pathway, which is regulated through the cytoplasmic ADAR1 p150 isoform [62, 74]. In the presence of ADAR1, endogenous dsRNAs are edited and considered as self dsRNAs that do not initiate immunogenic MDA5 recognition. In the absence or mutation of ADAR1, endogenous dsRNA is recognized by MDA5 as non-self, and then activates MAVS dependent phosphorylation of IFN regulatory factor 7 (IRF7) and IFN induction, which may lead to the pathogenesis of autoimmune diseases [76]. The interferon-induced isoform ADAR1 p150 may utilize its properties of specific binding to targets, such as through cytoplasmic localization of ADAR1 p150, and the Zα domain has become a key protein in the immune response [62, 77, 78].

ADAR1 p150 inhibits not only the MDA5/MAVS axis but also the protein kinase R(PKR) pathway. And eIF2a is phosphorylated following PKR activation by dsRNAs, which in turn shuts down cellular protein translation. Therefore, PKR also acts as another antiviral dsRNA sensor [79]. The level of PKR phosphorylation was increased after ADAR1 deletion, and the change was more pronounced after ADAR1 p150 knockdown [80]. In tumor cells, deletion of PKR (an IFN-inducible RNA recognition molecule) abrogated the IFN-induced growth inhibition phenotype in ADAR1-null tumor cells. Mechanistically, PKR misidentifies the unedited double-stranded RNA produced by ADAR1-null, resulting in a series of downstream stress responses and growth inhibition. The team also found that the RNA recognition molecule MDA5 was required to enhance inflammation and immune cell infiltration in Adar1-null tumors and that knockdown of both PKR and MDA5 deprived Adar1-null tumor cells of their enhanced responsiveness to immune checkpoint blockade (ICB) [80, 81].

Recently, three studies published in Nature have identified ZBP1 as a crucial effector in inducing inflammatory transcription due to ADAR1 mutation. ZBP1 recognizes double-stranded RNA derived from endogenous *Alu* elements. Pasparakis et al. found that ZBP1 can promote IFN activation and pathological progression in Adar1^mZα/-^ mice through a mechanism independent of RIPK1, RIPK3, MLKL-mediated necrosis, and caspase-8-dependent apoptosis [41]. Nicholas et al. have shown that the pathology caused by changes in the Z-DNA binding domain (ZBD) of ADAR1 is driven by the activation of ZBP1 [82]. Zhang Ting’s team identified ZBP1 mediated necrosis as a new determinant of tumor immunogenicity masked by ADAR1. Moreover, the therapeutic activation of ZBP1 induced necrosis provides a clearer pathway for reigniting the immune reactivity of human cancers with ICB [42].

In 2017, Nishikura’s team reported that ADAR1 p110 acts as a stress-responsive protein that rescues cells from RNA editing function independent of their survival. When cells are exposed to stress conditions, such as UV irradiation, ADAR1 p110 transiently translocates from the nucleus to the cytoplasm and protects specific anti-apoptotic mRNAs, a process that protects cells from stress-induced apoptosis and survival [83]. Staufen1 is a protein whose binding leads to the recruitment and subsequent mRNA degradation of the RNA helicase Upf1, called Staufen1-mediated mRNA degradation (SMD). By binding to the 3’-UTR, ADAR1 p110 competitively inhibits Staufen1 and prevents SMD. This isoform has an anti-apoptotic effect in inhibiting SMD in response to cellular stress [83, 84]. (Fig. 2A)

#### Aging

It has been shown that single nucleotide polymorphisms of ADARs are associated with extreme aging in humans. In model organisms, deletion of a single *Adar* gene in Drosophila leads to age-dependent phenotypes, such as neurodegenerative defects that are associated with dysregulation of the autophagic pathway. This disorder can be rescued by catalyzing inactive *Adar* mutants [85, 86]. Furthermore, inactivation of ADAR1 and ADAR2 in nematodes leads to a shortened lifespan [87], and downregulation of ADAR1 expression was found in the brain tissue of aging mice [63]. Genetic studies in model organisms clearly establish the role of ADARs in the regulation of lifespan and age-related diseases. Recent studies have shown that ADAR1 is significantly downregulated in senescent cells and that ADAR1 mutations can drive cellular senescence. The team found that ADAR1 downregulation promotes senescence through upregulation of p16^INK4a^ expression independent of RNA editing. This provides a mechanistic link between ADAR1 and organismal senescence [63].

In conclusion, ADAR1 is gaining attention as an important marker in human aging and age-related diseases.

## Role of ADAR1 in stem cells

Adenosine to inosine (A-to-I) RNA editing is catalyzed by ADAR1 and significantly alters the cellular transcriptome. To date, millions of A-to-I RNA editing sites have been identified in the human transcriptome. Although its mechanism of action in stem cells remains unclear, the role of RNA modification in cell fate control has only begun to be appreciated, as transcriptional and epigenetic barriers have been extensively examined and identified [88–91]. (Fig. 2B)

### Maintenance of hematopoietic system homeostasis

ADAR1 is widely expressed in mammals, and it has a critical role in hematopoietic homeostasis maintenance. Knockout of ADAR1 leads to premature death of mouse embryos along with severe defects in definitive hematopoiesis, including erythroid and bone marrow/granulocyte progenitors [92, 93]. In addition, the RNA editing function of ADAR1 is required for the survival of hematopoietic progenitors in the adult hematopoietic system [94], and deletion of ADAR1 in HSC results in upregulation of type I and type II interferon-inducible genes and rapid apoptosis of HSCs. Jamieson’s team reported that ADAR1-induced hyperediting in normal human hematopoietic progenitors accelerates cell cycle transit by impairing miR-26a maturation [70]. In general, ADAR1 serves as a crucial controller of hematopoietic stem cell (HSC) maintenance. Its major function is to inhibit interferon (IFN) signaling, which protects organisms from the damaging effects of immune activation [36].

### Regulation of developmental, differentiation and tissue homeostasis

RNA editing is widely involved in stem cell differentiation and development. Research has found that the Alu sequences in undifferentiated human embryonic stem cells are highly edited by ADAR1 [95]. The deletion resulted in mouse embryos dying between the E11.5 and E12.5 stages, as evidenced by rapid structural disintegration of the liver [50]. In 2021, Jian Liu et al. systematically described the genomic distribution of RNA editing sites in four stages of human cardiomyocyte differentiation. The expression level of ADAR1 was found to affect the global number of adenosine-to-inosine (A-to-I) editing sites but not the degree of editing. They found that these RNA editing sites are associated with several congenital and noncongenital heart diseases, thereby highlighting the link between cardiomyocyte differentiation and heart disease from an RNA editing perspective [96].

ADAR1 can affect the differentiation and neural induction of human embryonic stem cells (hESCs). Lingling Chen and Li Yang found that inhibiting the expression of ADAR1 changed the expression of important mRNAs and miRNAs in the directed neural differentiation of hESCs, and this functional change did not depend on the editing enzyme activity of ADAR1 [97]. Rather, it is determined by the potency of the RNA-binding domain of ADAR1[97]. Among them, the reduction in ADAR1 does not affect the stemness of human embryonic stem cells but reduces their ability to differentiate into neurons. ADAR1 can also regulate differentiation and neural induction through regulatory microRNA processing [97] .

In addition, ADAR1 regulates the development of skeletal muscle, despite the lower editing rate in skeletal muscle when compared to other tissues [32, 98]. Tan BC et al. reported that ADAR1 and miR-1/206 interact to control scheduled myoblast-myotube transition [98]. Recently, scientists have found that regulating A-to-I RNA editing ,which occurs at the U1 snRNA binding site at the 5 ‘splice site (5’ SS) of the *Alu* exon, control selenoprotein expression during skeletal myogenesis [99]. Furthermore, ablation of ADAR1 also impairs the cellular function of osteoblasts [100] and reduces bone mass in mice. In the intestinal microenvironment, ADAR1 deficiency promotes endoplasmic reticulum (ER) stress and interferon (IFN) signaling, induces inflammation, and leads to rapid apoptosis and loss of stem cells in the enteric and colon [101].

In conclusion, ADAR1 is essential for normal development and plays a key role in the maintenance of tissue homeostasis and stem cell differentiation.

### Impact on cell fate

There is plasticity in cell fate, and ADAR1 also plays a compelling role as a posttranscriptional modifier in the regulation of cell fate. First, ADAR1 is involved in regulating the reprogramming of human fibroblasts into induced pluripotent stem cells (iPSCs) [102]. Next, Miguel Fidalgo et al. showed that loss of ADAR1-mediated A-to-I editing disrupts the mesenchymal-to-epithelial transition (MET) and hinders the acquisition of induced pluripotency during iPSC reprogramming [103]. Namely, ADAR1 functions to induce reprogramming protection during MET. In addition to affecting cell reprogramming fate, ADAR1 deletion in human iPSCs directly promotes caspase-3-mediated apoptosis, thereby altering cell fate [104].

Another team found that ADAR1 is the main RNA editing enzyme dysregulated in glioblastoma stem cells (GSCs) [105]. The upregulation of ADAR1 promotes GSC self-renewal and stemness maintenance. They also found that ADAR1 inactivity or the inhibition of tyrosine kinase 2 (TYK2), blocking the upstream JAK/STAT pathway, impaired GSC self-renewal and stemness [105]. This finding also demonstrates that specific small-molecule inhibitors targeting the ADAR1 pathway can effectively block the self-renewal and stemness of GSCs, thus suggesting a potential therapeutic strategy for glioblastoma.

Taken together, ADAR1 and its A-to-I editing activity play a key role in cell fate transition. This shows that ADAR1 affects stem cell function in both physiological processes and pathological conditions, which will help to gain a more in-depth and refined understanding of epigenetics and develop innovative targeted therapeutic strategies.

## Role of ADAR1 in remodeling the stem cell-related tumor microenvironment

The tumor microenvironment (TME) refers to the close relationship between the occurrence, growth and metastasis of tumors and the internal and external environment in which the tumor cells are located. ADAR1 has been found to be upregulated in a variety of human and animal cancers. Among them, the upregulation of ADAR1 is closely related to the occurrence, development and prognosis of cancer, including breast cancer, liver cancer, lung cancer, esophageal cancer, prostate cancer, chronic myeloid leukemia and multiple myeloma [106–110].

Consistent with the elevated expression of ADAR1, the editing levels of its substrates have also been markedly elevated in various stem cell-related cancers. In relapsed multiple myeloma (MM), glioma-associated oncogene 1 (GLI1) upon ADAR1 editing can promote the activation of the Hedgehog signaling pathway and the self-renewal of stem cells by stabilizing their own transcriptional processes [111]. In addition, ADAR1-mediated hyperediting antizyme inhibitor 1 (AZIN1), which is capable of influencing self-renewal and differentiation at the stem cell level, has been discovered in hepatocellular carcinoma (HCC) and colorectal and gastric cancers [109, 112–114]. After editing, AZIN1 has a higher affinity for antienzymes, thereby inhibiting its degradation of growth-promoting proteins [115], allowing cells to pass the G1/S phase checkpoint, and the degree of malignancy is greatly increased [109]. Dr. Catriona Jamieson et al. found that cancer stem cells (CSCs) incorporate an RNA editing system for self-proliferation [52]. Jamieson’s team identified ADAR1’s role in editing stem cell regulatory let-7 microRNAs; this process drives leukocyte precursors to transform into leukemia stem cells and promotes leukemia stem cell proliferation [51]. In another study, miR-200b was found to be impaired in its ability to inhibit the epithelial-mesenchymal transition (EMT) regulator ZEB1/ZEB2 primarily due to upregulated RNA editing via ADAR1, but instead, it altered the inhibitory target leukemia inhibitory factor receptor (LIFR), promoting cell invasion and migration [116]. However, in contrast to the cases described above, in metastatic melanoma and aggressive breast cancer cells, ADAR1 silence or deletion can also control oncogenic or suppress the biogenesis of tumor miRNAs to enhance their malignant properties [117–120].

In summary, upregulation of ADAR1 in the tumor microenvironment affects changes in its components that regulate tumor growth and migration.

## Strategies and challenges of targeting ADAR1 in cancer immunotherapy

**Fig. 3:**
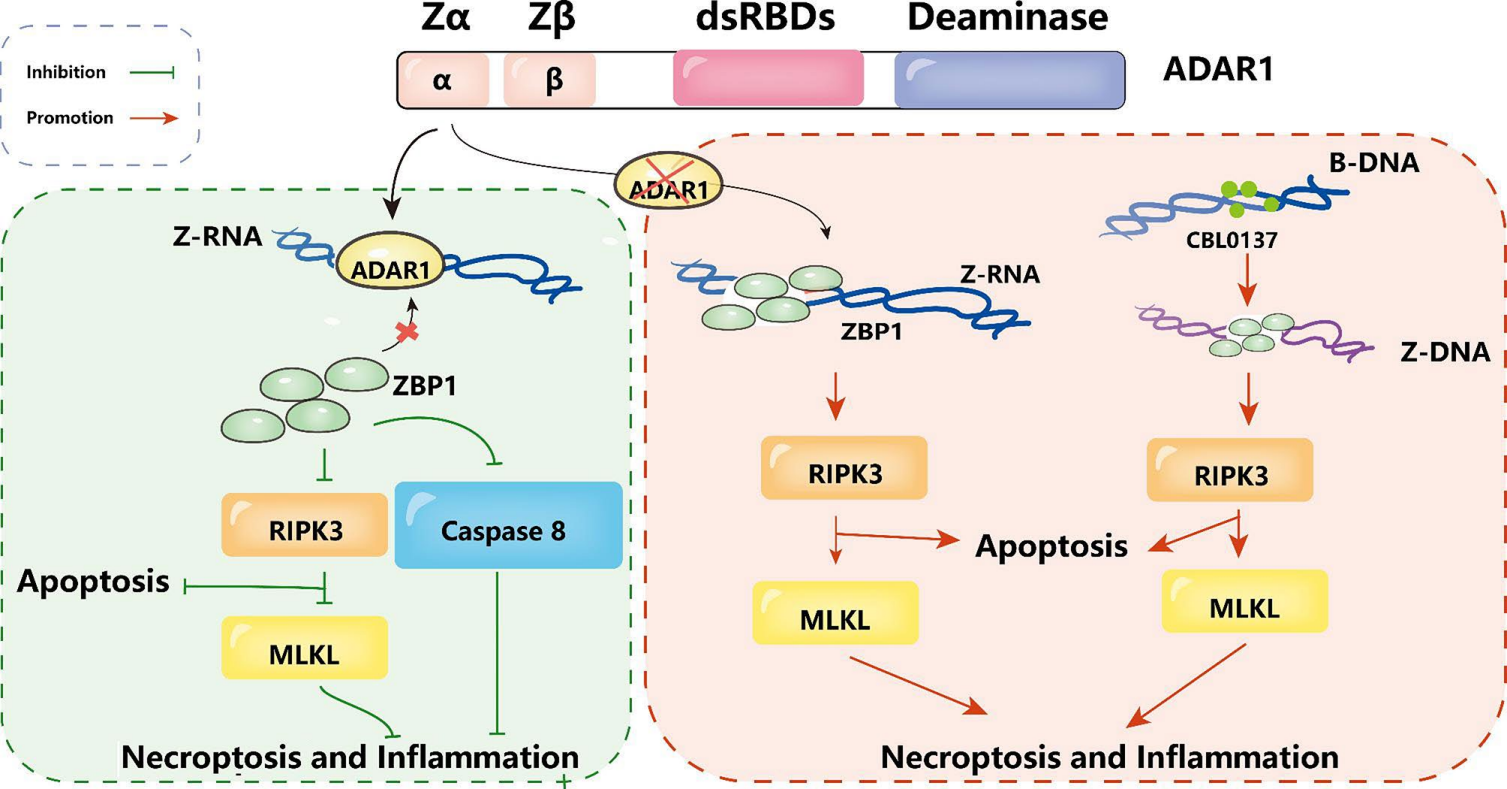
Function of ADAR1 Zα. ADAR1 can promote A-to-I editing of endogenous Alu elements through the Zα structural domain to prevent dsRNA formation by reverse Alu repeat pairing. ADAR1, if absent, will induce ZBP1 activation, which in turn triggers caspase-8-dependent apoptosis and MLKL-mediated necroptosis of ADAR1-deficient cells. The small molecule compound CBL0137 can specifically induces Z-DNA conformation of genomic sequences, thus inducing ZBP1-dependent cell death

Since ADAR1-mediated posttranscriptional adenosine-inosine RNA editing promotes cancer progression and treatment resistance, the immune function of ADAR1 remains important when discussing cancer therapy. Immunogenic dsRNAs are able to trigger interferon-dependent antitumor responses by activating A-type double-stranded RNA (A-RNA)-sensing proteins [121]. It has also been previously reported that loss of function of ADAR1 can sensitize cancer stem cells to immune checkpoints by increasing the levels of these double-stranded RNAs (dsRNAs) [80, 122]. In a syngeneic mouse melanoma model, ADAR1 deficiency leads to the infiltration of CD8^ +^ T cells in this tumor microenvironment and the expression of chemokines associated with T-cell and natural killer cell recruitment [80]. In hematopoietic malignancies, when gene expression profiles of normal, chronic, and sequentially transplanted acute crisis chronic myeloid leukemia (CML) progenitors were compared, it was found that IFN-γ pathway gene expression was increased, along with enhanced ADAR1 p150 expression and increased adenosine-inosine RNA editing during CML progression [52, 70, 123]. In addition, editing of GLI1 by ADAR1 resulted in an increased proportion of drug-resistant cells in myeloma, suggesting that inhibition of GLI1 editing by ADAR1 could improve the sensitivity of myeloma cells to drug therapy [111, 124]. The above phenomena indicated that ADAR1 can be targeted as an immune checkpoint for hematopoietic lineage malignancies. Unfortunately, there are no drugs specific for ADAR1 to achieve immune checkpoint blockade. However, some researches spot that ADAR1 could inhibit ZBP1-mediated apoptosis, which means Zαdomain of ADAR1 can inhibit ZBP1 by recognizing dsRNA, restraining caspase-8-dependent apoptosis and MLKL-mediated necroptosis [43, 82]. Zhang et al. showed that overexpression of ADAR1 can curb ZBP1 activation through Z-RNA editing, repressing ZBP1-mediated programmed cell necrosis and tumor immunity activation [42]. Combined with previous findings, it is surprising that Zhang et al. identified CBL0137, a small molecule, that induces the production of Z-DNA to trigger ZBP1-dependent cell death [42]. (Fig. 3)

In addition, it has been reported in the literature that Myc is consistently upregulated in pancreatic, gastric and breast cancers. Also, the higher the expression of ADAR1, the higher the resistance of pancreatic cancer cells to BET inhibitors by stabilizing c-Myc. These findings open the possibility of using a combination of BET and ADAR1 inhibitors as the optimal treatment for pancreatic cancer [125].

## Conclusions and perspectives

Today, millions of A-to-I RNA editing sites have been identified in the human transcriptome, but the functions of most RNA editing events are unknown. Understanding the molecular mechanisms by which RNA editing affects gene expression and phenotype may be promising. This will help researchers unravel the genetic mysteries behind many human diseases [126–129]. The RNA editing function of ADAR1 has been transformed by scientists into a tool for the treatment of genetic diseases or in scientific research [130–132]. For example, genome editing is a method suitable for in vitro research, and it may be suitable for editing fertilized eggs. However, genome editing is still not applicable to the human embryos due to ethical limitations [131, 133]; instead, the changes produced by RNA editing are not permanent because they do not affect the body’s genome sequence and can be done in a sequence-specific manner [129]. Therefore, RNA editing is preferable to genome editing for therapeutic purposes. Human-directed RNA editing is an important technique that can repair genes and ultimately regulate the function of encoded proteins. RNA editing modifies the genetic code of transcripts to enable the treatment of genetic disorders [134]. Zhang Feng’s team developed a strategy called RESCUE (RNA Editing for Specific C to U Exchange, C to U exchange-specific RNA editing), which expands the range that CRISPR tools can target, including modifiable positions in proteins points, such as phosphorylation sites [135]. These sites act as on/off switches for protein activity and are primarily found in signaling molecules and cancer-related pathways.

In summary, ADAR1-mediated RNA editing is required for stem cell fate and function, and targeting this pathway has been proposed as a new therapeutic strategy in cancer. However, exploring the role of A-to-I editing in both normal and malignant states is essential for understanding the potential consequences for therapeutic intervention. Based on the clinical application value of ADAR1, we propose the following considerations: (1). Studies have shown that ADAR1 can not only regulate mRNA expression but also affect miRNA expression. As miRNA is an important component of stem cell exosomes, the role and mechanism of ADAR1 in exosome driving effect is worthy of further study. (2). With the deepening of research, many functions of ADAR1 have been found to not depend on RNA editing enzyme activity, which means that the function of ADAR1-independent editing enzyme activity in stem cells still needs to be further developed. (3). ADAR1 is a potential target for cancer therapy, and the development of ADAR1 inhibitors based on CSCs will be another strategy for tumor therapy. Targeting ADAR1 at the same time will cause autoimmune disease, which may be a serious side effect. (4). It is exciting to explore RNA editing as a promising avenue. The identification and characterization of appropriate biomarkers to evaluate the efficacy of RNA editing targeted approaches in different subtypes of patients with stem cell-related diseases will help guide therapeutic development.

## Data Availability

Not applicable.

## Abbreviations

ADAR: Adenosine deaminase acting on RNA
AGS: Aicardi-Goutières syndrome
A-RNA: A-type double-stranded RNA
ASC: Adult stem cells
AZIN1: Hyper-editing antizyme inhibitor 1
CML: Chronic myeloid leukemia
CSC: Cancer stem cells
DSH: Dyschromatosis symmetrica hereditaria
dsRNA: Double-stranded RNA
EMT: Epithelial-mesenchymal transition
ER: Endoplasmic reticulum
ESC: Embryonic stem cells
GLI1: Glioma-associated oncogene 1
GSCs: Glioblastoma stem cells
HCC: Hepatocellular carcinoma
hESC: Human embryonic stem cells
HSC: Bone marrow hematopoietic stem cells
IFN: Interferon
iPSC: Induced pluripotent stem cells
ISG: Interferon-stimulated genes
ISRE: Interferon-stimulated response element
LSC: Leukemia stem cell
BC-LSC: Blast crisis leukemia stem cell
LIFR: Leukemia inhibitory factor receptor
ICB: Immune checkpoint blockade
MET: Mesenchymal-to-epithelial transition
miRNA: MicroRNA
MM: Multiple myeloma
MSC: Mesenchymal stem cells
NES: Nuclear export sequence
NFYA: Nuclear transcription factor Y subunitα
NLS: Nuclear localization sequence
RESCUE: RNA Editing for Specific C to U Exchange, C to U exchange-specific RNA editing
SMD: Staufen1-mediated mRNA degradation
TME: The tumor microenvironment
TYK2: Tyrosine kinase 2
ZBP1: Z-DNA binding protein 1
